# The Discovery of Novel Genomic, Transcriptomic, and Proteomic Biomarkers in Cardiovascular and Peripheral Vascular Disease: The State of the Art

**DOI:** 10.1155/2016/7829174

**Published:** 2016-05-19

**Authors:** Stefano de Franciscis, Laurent Metzinger, Raffaele Serra

**Affiliations:** ^1^Interuniversity Center of Phlebolymphology (CIFL), International Research and Educational Program in Clinical and Experimental Biotechnology, Headquarters, University Magna Graecia of Catanzaro, Viale Europa, 88100 Catanzaro, Italy; ^2^Department of Medical and Surgical Sciences, University of Catanzaro, Viale Europa, 88100 Catanzaro, Italy; ^3^C.U.R.S, Laboratoire INSERM U1088, Chemin du Thil, Université de Picardie Jules Verne, 80025 Amiens Cedex 1, France

## Abstract

Cardiovascular disease (CD) and peripheral vascular disease (PVD) are leading causes of mortality and morbidity in western countries and also responsible of a huge burden in terms of disability, functional decline, and healthcare costs. Biomarkers are measurable biological elements that reflect particular physiological or pathological states or predisposition towards diseases and they are currently widely studied in medicine and especially in CD. In this context, biomarkers can also be used to assess the severity or the evolution of several diseases, as well as the effectiveness of particular therapies. Genomics, transcriptomics, and proteomics have opened new windows on disease phenomena and may permit in the next future an effective development of novel diagnostic and prognostic medicine in order to better prevent or treat CD. This review will consider the current evidence of novel biomarkers with clear implications in the improvement of risk assessment, prevention strategies, and medical decision making in the field of CD.

## 1. Introduction

Cardiovascular disease (CD) and peripheral vascular disease (PVD) are leading causes of mortality and morbidity in western countries. CD and PVD impose also a huge burden in terms of disability, functional decline, and healthcare costs. CD includes ischemic heart disease, hypertension, heart failure, and cerebrovascular disease and PVD includes arterial and venous disease [1–3].

In the last decades great progresses have been made in the treatment of these diseases; nevertheless, mortality and morbidity remain high, and trusting only in cardiovascular risk factors knowledge, we are also unable to effectively predict which subjects are at real risk of getting these kinds of health problems [4].

For these reasons, great efforts have been made, in recent years, to identify several biomarkers with diagnostic and prognostic implications as well as for primary or secondary prevention purpose [5].

In the postgenomic era in biomedical research, the biomarker research focused on the role of genes (genomics) including the understanding of gene transcriptional regulation (transcriptomics), the biochemical functions of all the gene products and their interactions (proteomics), and learning how they influence cellular biochemistry, metabolism, and disease development [6, 7].

The aim of this review is to summarize the most frequently studied novel biomarkers with clear implications in the improvement of risk assessment, prevention strategies, medical decision making, and clinical outcomes and also in their ease of use in daily clinical practice.

## 2. Literature Search

Based on the large amount of evidences existing in the field of biomarkers, we decided to search for relevant articles in three main areas of interest: genomics, proteomics, and transcriptomics in CD and PVD. PubMed, Scopus, and ScienceDirect databases were used for the search strategy.

## 3. Genomic Biomarkers

Recent genome-wide association studies (GWAS) identified several well-replicated single-nucleotide polymorphisms (SNPs) associated with coronary artery disease (CAD), but only modest improvements in CAD risk prediction were actually seen through the use of genetic risk scores based on multiple SNPs. Therefore, it has been suggested that many more SNPs are needed to effectively identify a substantial genetic risk for CAD [8–10].

Heritability of hypertension ranges from 31% to 68% and there is also an evident difficulty in identifying genes for this condition as a large number of candidate genes have been tested for association with hypertension without convincing results, with a reported 50% survival four years after diagnosis [11].

Heart failure (HF) has important genetic implications. Actually, while HF is often caused by CAD, hypertension, diabetes, and valvular heart disease, several studies suggested that the risk of HF depends also on genetic predisposition but due to the highly variable expressivity and penetrance of genetic alterations, this field needs further investigations [12].

Some studies investigated carotid artery atherosclerosis (CAA) from a genetic point of view: Lan et al. [13] studied the relationship between polymorphisms in the macrophage migration inhibitory factor (MIF) gene and the severity of CAA in a cohort of Taiwanese patients with ischemic stroke. MIF is a cytokine that was originally isolated from T lymphocytes and identified as an inhibitor of the random migration of macrophages and it is also constitutively expressed by smooth muscle cells and endothelial cells in normal blood vessels. The study demonstrated that polymorphisms in the MIF gene promoter were associated with CAA severity in ischemic stroke patients and these genetic variants may serve, in the next future, as markers for an individual's susceptibility to CAA.

Biscetti et al. [14] investigated the distribution and the interaction between gene polymorphisms encoding proinflammatory molecules in a population with internal carotid artery stenosis (ICAS). They found that the following genes and their variants, interleukin- (IL-) 6 (IL-6), IL-1*β*, monocyte chemoattractant protein-1 (CCL2), macrophage inflammatory protein-1*α* (MIP-1*α*/CCL3), E-selectin (SELE), intercellular adhesion molecule 1 (ICAM1), and Matrix Metalloproteinase-3 (MMP-3) and MMP-9, were independently and significantly associated with ICAS.

Family studies show that heritability of peripheral arterial disease (PAD) ranges from approximately 20% to 45% after adjusting for atherosclerotic risk factors but to date no definitive genetic markers have been identified for PAD. A relatively small number of case-control studies have assessed the associations of specific gene polymorphisms with the presence of PAD. However, results coming from these studies have not established a consistent genetic marker for PAD [15, 16].

Genetic influences may play a crucial role in the onset of arterial aneurysm, and the most genetically studied region of aneurysm formation is the thoracic aorta where several genes have been identified as predisposing factor for aneurysm and also dissection.

For thoracic aneurysms and dissection, there is some evidence that, despite predicting the risk for the onset, routine genetic screening may also provide information that enables genetically personalized care for affected patients. Genetic alterations of Transforming Growth Factor (TGFB), Transforming Growth Factor Beta Receptor 1 (TGFBR1), TGFBR2, small mother against decapentaplegic 2 (SMAD2), and Transforming Growth Factor Beta 2 (TGFB2) genes seem to be pivotal, as they may be responsible for both syndromic and nonsyndromic ascending thoracic aortic aneurysm and dissection [17].

For abdominal aortic aneurysm, a recent systematic review and meta-analysis [18] evidenced how in the last years, due to uncorrected multiple testing and flexible study design, several apparently false associations have been reported. This article showed several supported associations for lipoprotein metabolism genes polymorphisms such as low-density lipoprotein receptor-related protein 1 (LRP1), low-density lipoprotein receptor (LDLR), Sortilin 1 (SORT1), and lipoprotein(a) (LPA). Other gene polymorphisms, such as the ones found for interleukin-6 receptor (IL6R), Matrix Metalloproteinase-3 (MMP-3), Angiotensin II Receptor, and Type 1 (AGTR1), seem to be also involved.

While several genetic models have been studied and proposed to explain the heritability of intracranial aneurysms (IAs), at the moment, no diagnostic test based on genetic knowledge is currently available to effectively identify patients who are at higher risk for developing IAs [19].

On the venous side, recent evidences showed that the gene polymorphisms prothrombin (factor II) G20210A and factor V Leiden not only increase the already well-known risk for venous thromboembolism (VTE) deep vein thrombosis (DVT) and pulmonary embolism (PE) but also raise the risk for cerebral venous thrombosis, a stroke subtype that is particularly more common in women, especially of reproductive age [10, 20].

Chronic Venous Disease (CVD), Varicose Veins (VV) and its more severe manifestation, Chronic Venous Insufficiency (CVI), with its main complication, and Chronic Venous Ulceration (CVU) were widely studied: Serra et al. showed that a functional variant of FOXC2 gene may account for a predisposition for VV [21].

A recent study demonstrated that regulatory genes of arachidonic acid metabolism and mediators of the inflammatory reaction, hydroxyprostaglandin dehydrogenase-15 (HPGD), are overexpressed in CVI patients. The study also showed that regulatory genes of collagen production (collagen type 13*α*1 and collagen type 27*α*1 genes) were downregulated in veins affected by superficial reflux disease [22].

Jin et al. showed the association between an insertion and a deletion polymorphism (rs3917) within the 3′ untranslated region of COL1A2 (alpha-2 type I collagen gene) causing a genetic variation in the COL1A2 gene that seems to influence the possibility of developing CVI [23].

A further candidate gene which was studied is thrombomodulin, a marker of endothelial injury [24, 25]. The hemochromatosis C282Y gene mutation has been associated with the onset of CVU by Zamboni et al.; in fact this group showed how this mutation was more frequently associated with skin changes in patients with CVI [25, 26]. While these studies are promising, the samples sizes were small, the studies were unpowered, and they lack also replication and, therefore, the area of the genetic basis of venous disease needs further investigation [25].

## 4. Transcriptomic Biomarkers

Since several decades, the clinician has access to numerous tools for diagnosis and therapeutic follow-up of most known pathologies. In today's medicine, progress in molecular biology techniques allows studying now in one single experimentation a given family of molecules. Transcriptomics is the technique that allows the study of RNA. The most studied RNA group is represented by the messenger RNAs (mRNAs) which are defined as ribonucleotide sequences complementary of the coding strand of the DNA genes. In this instance, the RNA group is called the transcriptome. However, there exists other RNA types in the cell, such as ribosomal RNAs which constitute 80% of total RNAs and are instrumental for translation of mRNA sequence into proteins. A whole wealth of other noncoding RNAs has also appeared in the last 15 years and we will discuss their use later in this chapter.

The cDNA microarrays allow simultaneous measurement of several tens of thousands of genes in a given sample using hybridization of retrotranscribed RNA. The resulting cDNAs are labelled and amplified on specific probes fixed to the array (also known as chip or biochip). These studies are usually performed by comparing the transcriptome of two populations (typically, a control, healthy group is compared to a diseased group) to detect a signature of genes specific for a given disease. Cyanide dyes, such as Cy3 and Cy5, are used to give to each sample a specific color. Differential expression is analyzed using different statistical analysis processes.

Early transcriptome analyses have established catalogs of genes expressed preferentially in specific cells or tissues. To cite one of the most ancient studies, Piétu et al. have published the existence of genes expressed only in skeletal muscle [27]. Usual applications include also the measurement of kinetic response to a specific effector/drug and the clustering analysis of genes responding with a similar pattern. Transcriptomic studies can also permit the characterization of regulatory pathways in given tissues, at various stages of development. These expression profiles can, in turn, help to establish a “gene signature,” characteristic for a pathological sample, such as a tumor. Transcriptomics can also be useful to provide prognostic predictor values. Expression profiles, for this instance, are derived from the comparison of two groups of patients sharing several criteria but different in one specific clinical feature, such as, for example, hypertensive and normotensive state. In fact, comparing the two groups will help identify genes differentially expressed between the two groups to establish a prognostic signature.

In the first effort to obtain a global portrait of gene expression in the failing heart, Barrans and colleagues [28] constructed a human cardiovascular cDNA microarray containing approximately 10 000 genes and selected 38 genes showing a minimum of twofold differential expression, in the diseased tissue. Gaertner et al. [29] performed an elegant transcriptomic study that compared the transcriptomes of myocardial samples taken from the left ventricle (LV) and right ventricle (RV) of 13 terminal cardiomyopathy patients from their heart-transplantation program. Of these, six had arrhythmogenic right ventricular cardiomyopathy, whereas seven had idiopathic dilated cardiomyopathy, without signs of familial etiology or coronary artery disease. Furthermore, they compared the data with six nonfailing donor hearts. They used the Affymetrix HG-U133 Plus 2.0 arrays to provide coverage of the whole human genome with 47,000 transcripts and variants of 38,500 characterized human genes and 54,000 probe-sets. They found that only ~15-16% of the genes was commonly regulated in the failing myocardium compared to nonfailing samples. In addition, arrhythmogenic right ventricular cardiomyopathy and idiopathic dilated cardiomyopathies were clearly distinct on the transcriptome level. Comparison of the expression patterns between the failing RV and LV using a paired *t*-test revealed a lack of major differences in ARVC (arrhythmogenic right ventricular cardiomyopathy) hearts. Note that this transcriptomic study on myocardial samples may provide important information on the changes occuring in diseased hearts but is not useful in predicting susceptible individuals but may still give clues to predict more precisely susceptible individuals. Huan et al. [30] performed a meta-analysis of results deriving from six studies of transcriptomic profiles of blood pressure (BP)/hypertension in the blood of 7017 individuals not receiving antihypertensive drug treatment. Thirty-four genes, mostly involved in inflammatory response and apoptosis pathways, were found to be differentially expressed in relation to BP, including FOS, a leucine-zipper transcription factor, and PTGS2, implicated in prostaglandin biosynthesis, that were already involved in hypertensive processes. Authors estimated that their analysis could explain 5%–9% of interindividual variance in BP.

Transcriptomics can also help to investigate the gene regulation involved in therapeutic approaches. Lam et al. [31] looked at the effect of six-month calorie restriction (CR) and/or structured exercise on the transcriptome of abdominal subcutaneous fat in a cohort of 24 overweight patients. They found that CR elicited more regulatory changes (88 genes impacted, including 27 transcription/translation regulators interestingly) than structured exercise (39 genes impacted, including only one transcription/translation regulator). This may explain at least in part the preponderant effect of CR on delaying primary aging. Others have looked at the roles of differentially expressed mRNAs in venous diseases. Zhou et al. [32] have used microarray approaches to explore the molecular mechanisms and potential biomarkers of single venous thromboembolism (SVT) and recurrent venous thromboembolism (RVT) in the blood. They found that at least 22 mRNAs were deregulated. They were implicated in translational elongation, cell proliferation, ribosomal pathways, negative regulation of heart contraction, and protein export. The authors concluded that all these genes are potential biomarkers of SVT and RVT. Once relevant genes are identified using systemic approaches, one can look at polymorphisms of their sequences and study the relevant differences between patients and healthy individuals. For instance, Deser et al. [33] have studied polymorphisms of IL-18 in peripheral blood mononuclear cells from peripheral artery disease patients. They found that IL-18 mRNA levels may be an important marker for peripheral artery occlusive diseases, as they are correlated with several biochemical markers such as triglyceride and cholesterol levels.

These are, of course, only representative examples of the numerous studies using microarrays in the cardiovascular field (a PubMed search using the terms “cardiovascular + microarray + transcriptome” brings 249 hits). However, it is important to note that the main current flaw in the microarray technique is that it is difficult to compare data between studies due to the lack of standardization in platform fabrication, assay protocols, and analysis methods. Also, since most of the findings are recent, the various mRNAs described in this review have not yet been reproduced by multiple researchers and further work will be clearly needed to establish if they may function as effective biomarkers in the CD field.

A growing number of publications show that microRNAs (miRNAs) are new solutions to identify biomarkers in the serum and the pathological tissues. A vast number of them use RNA-based microarray approaches specifically developed for these small RNAs, with the generic term of RNomics. The miRNAs are short noncoding RNA of 18–24 nt. This class of small RNA is involved in regulating gene expression at the posttranscriptional level by translational repression or degradation of the target mRNA. The miRNAs regulate several biological processes and then are implicated in several pathologies. As an example, Li et al. [34] have shown that a signature of seven miRNAs is sufficient to predict overall survival and survival without relapse in a Chinese population suffering from stomach cancer. Numerous studies demonstrate an alteration of miRNA expression in CD [35], particularly in the serum. Indeed, Mitchell et al. [36] demonstrated, for the first time, the presence of circulating miRNA in human plasma. This discovery was surprising as plasma is known to have high RNase activity [37]. These circulating miRNAs have many origins and way of transport; they could be released from cells by passive leakage due to injury or chronic inflammation (in this case the way of transport is unknown); the miRNAs could be also released by active secretion via cell-derived membrane vesicles or released by a protein miRNA complex like argonaute 2, nucleophosmin-1 (NMP1), or lipoprotein (HDL). Cell-derived membrane vesicles and ribonucleoproteic complex confer the stability and the protection against RNase activity to these circulating miRNAs. Many studies have demonstrated a correlation between plasma miRNA and diseases, establishing them as potential noninvasive biomarkers of several pathologies mainly in cancer and cardiovascular diseases [38]. Concerning CD, an early increase of miR-208a, miR-133, and miR-1 is observed in plasma of patients suffering of myocardial infarction [39]; in essential hypertension miR-let-7e is increased and miR-296-5p is decreased in patients' plasma [40].

Patients with coronary artery disease or diabetes display reduced levels of endothelial-enriched miRNAs, such as miR-126 [41]. Embolization of carotid stenotic plaques is the direct cause of stroke in nearly 20% of cases. The genetic mechanisms and especially the roles played by miRNAs in the regulation of plaque destabilization and rupture are mostly unknown. We compared the expression of seven miRNAs allegedly involved in plaque growth and instability between symptomatic and asymptomatic human carotid plaques. Six miRNAs were significantly overexpressed in symptomatic versus asymptomatic plaques, and one (miR-125a) expression was significantly inversely correlated with the circulating level of low-density lipoprotein cholesterol in the symptomatic group. This suggests a potential regulatory role for these miRNAs in evolution of the plaque towards growth, instability, and rupture. Studies based on larger sample sizes are required to determine the potential use of miRNAs as biomarkers or therapeutic targets for stroke [42]. AF is a complex arrhythmic atrial disease with important cardiovascular and systemic consequences. Sardu et al. [43] have studied the potential involvement for miRNAs to be biomarkers for their changes after atrial fibrillation catheter ablation and have shown that several miRNAs are deregulated, opening new perspectives for clinical evaluations and therapeutic management strategies in the context of an arrhythmic disease. The miRNAs are also important as novel biomarkers for arterial aneurysms and peripheral artery diseases. Zhang et al. have measured miRNAs in plasma samples from 10 abdominal aortic aneurysm (AAA) patients and 10 healthy controls by microarray and confirmed the most differentially expressed miRNAs in a training cohort of 120 subjects, including 60 AAA patients and 60 normal controls [44]. They concluded that miR-191-3p, miR-455-3p, and miR-1281 are the most useful diagnosis biomarkers for AAA. On the other hand, Stather et al. [45] used whole-blood from 15 AAA male patients and ten healthy volunteers and confirmed the results using RT-q-PCR in peripheral blood and plasma samples from a cohort of 120 patients. They found that the most deregulated miRNAs in their hand were let-7e, miR-15a, miR-196b, and miR-411. These results and others are summarized in a recent review, focusing on miRNA expression modulation during AAA initiation and propagation [46]. Various efforts have been done to study the roles of differentially expressed miRNAs in venous diseases. We have shown that several miRNAs are dysregulated in the cerebral microvasculature in the CKD context [47], highlighting a possible role for these miRNAs in the appearance of cerebral strokes common in CKD patients. Very recently, a pilot study involving 20 patients suffering from unprovoked venous thromboembolism has shown that plasma levels of miR-103a-3p, miR-191-5p, miR-301a-3p, and miR-199b-3p were downregulated in plasma of patients, while the levels of miR-10b-5p, miR-320a, miR-320b, miR-424-5p, and miR-423-5p were upregulated.

Another category of players, a heterogeneous group of long transcripts that regulate gene expression at transcriptional and posttranscriptional levels called long noncoding RNAs, has now entered the field [48]. As is usually the case, lncRNA biomarkers were first described in studies performed in various cancers [49, 50]. However, such clinical studies were until recent times never performed in cardiovascular diseases. A recent study [51] indicated that the myocardial transcriptome is dynamically regulated in advanced HF and the expression profiles of lncRNAs can discriminate failing hearts of different pathologies. Using a microarray (arraystar lncRNA array) focused on 33,045 human lncRNAs, Kumarswamy et al. [52] studied the plasma RNA from patients with or without left ventricular remodeling after myocardial infarction. They identified the mitochondrial lncRNA LIPCAR as a potential biomarker of development in myocardial infarction patients, with additional association with cardiovascular death, independent of other predictors. It is now increasingly clear that lncRNAs have an important regulatory role in cardiac physiopathology and might be innovative biomarkers of cardiovascular disease development and prognosis [53].

## 5. Proteomic Biomarkers

Proteomics is the study of proteins expressed by a genome of an organism that are involved in both physiologic and pathologic conditions. Proteomic analysis may provide the opportunity to understand the pathophysiology of disease and it also may permit us to evaluate candidate biomarkers in tissues of human beings as well as to identify therapeutic targets and, thus, individualized therapies. Although recent research evaluated several systematic changes in protein expression in response to intrinsic or extrinsic perturbations in the field of cardiovascular disease, yet there is no enough validation for the majority of these candidates to effectively serve as sensitive and specific biomarkers for clinical application [54].

Based on the amount of evidences existing in the current and updated literature we selected those biomarkers dealing with the extracellular matrix (ECM) in cardiovascular tissues as the ECM constitutes more than half of the wall mass and vascular wall. Moreover, matrix protein synthesis and metabolism are strictly related to blood vessel structure, homeostasis, and functions. In fact, ECM is a highly dynamic structure that continuously undergoes remodeling mediated by several matrix-degrading enzymes, and, thus, variations in the composition and structure of ECM may affect the overall structure, biomechanical properties, and cell response of several organs and biological system. In this context, Matrix Metalloproteinases (MMPs) are a family of proteolytic enzymes that are regulated by inflammatory signals to mediate changes in ECM [55, 56].

Friese et al. identified several patterns of plasma MMPs in hypertension with overexpression of MMP-9 in the early manifestations of the disease and then elevation of MMP-2 and MMP-10 linked to target organ damage [57].

The progression of ventricular remodeling, after a myocardial infarction or other conditions, such as viral injury, is also mediated through MMP activation that subsequently causes the breakdown of the collagen and the elastin framework. This process leads to ventricular hypertrophy and dilation with subsequent congestive heart failure [58].

There is increasing evidence that alterations in the MMPs regulation and expression may be directly implicated in arterial and venous disease. Significant progress has been made in identifying the changes in the levels and activity of MMPs in several physiological and pathological conditions [59].

Increased activity and/or loss of control of MMPs have been related to vascular remodeling [60]. In fact MMPs play also a role in vascular smooth muscle (VSM) cell migration and neointima formation after localized vascular injury. MMPs may also promote aneurysm formation and aneurysm rupture. Varicose Veins development may be mechanistically similar to aneurysm development and MMPs may, in this way, contribute to vein wall weakness and subsequent vein dilation. The specific role of MMPs in venous ulcers pathogenesis remains unclear; however, MMPs hyperactivity and dysregulation seem to inhibit wound healing via excessive basement membrane degradation leading to loss of epidermal integrity [60–62]. In this context, MMP-1 and MMP-8 seem to be primarily involved with chronic or irreversible complications of vascular disease (such as difficult-to-heal or infected venous ulcers and postthrombotic syndrome) as some recent studies performed on plasma, wound fluid, and tissue sample concentrations demonstrated [63–65].

Recent studies have shown that MMP-9, an endopeptidase capable of degrading the extracellular matrix, is thought to be particularly associated with atherosclerosis and plaque rupture. Therefore, MMP-9 is considered to be an important mediator of vascular remodeling and plaque instability. The MMP-9 action is also enhanced by neutrophil gelatinase-associated lipocalin (NGAL), also known as lipocalin-2, a 25 kDa glycoprotein, that is, found in the granules of human neutrophils. In fact NGAL was shown to be able to bind and stabilize MMP-9 [66].

The formation of a complex with NGAL and MMP-9 seems to be crucial for atherosclerotic plaque erosion and thrombus formation and recent studies have postulated that this complex may be implicated in aneurysms rupture and in delayed wound healing of chronic venous ulcers as some recent studies performed on plasma and affected tissues levels demonstrated [67, 68]. A further recent experience showed that NGAL plasma levels were independently associated with increased risk of CVD mortality, all-cause mortality, and cardiovascular disease during long-term follow-up [69].

Increased plasma levels of MMP-9 and NGAL expression have been also related to deep vein thrombosis and pulmonary embolism [70].

MMP-9 and NGAL seem also to increase together the systemic inflammation which participates in atherosclerosis evolution from the early development of endothelial dysfunction, to formation of mature atheromatic plaques, to the ultimate endpoint, rupture, and thrombotic complications [66].

Thus, several studies into this field have shown that MMPs and NGAL may be released into the blood in sufficient quantities to act as diagnostic or prognostic markers for various clinical conditions [58].

Substrate specificity for the MMPs and for NGAL is not yet fully characterized. Additionally, several studies showed how MMPs may act cooperatively both in degrading ECM and in the various pathological processes. Therefore, more research is needed to identify specific MMPs patterns for each kind of related diseases.

There are also two other families of metalloproteinases, ADAMs (a disintegrin and metalloproteinases) and ADAMTSs (a disintegrin and metalloproteinases with thrombospondin motifs), which can be detected in the blood, and they seem to be involved in several mechanisms of cardiovascular and peripheral vascular disease [71, 72], especially in CVD evolution toward CVU where a recent study [71] identified high serum levels of ADAM-10, ADAM-17, and ADAMTS-4 to be responsible of the maintaining of chronic inflammation in patients with CVD, but their exact role remains to be further clarified.

There are also naturally occurring inhibitors of metalloproteinases, called Tissue Inhibitors of Metalloproteinases (TIMPs), which can partially or completely neutralize metalloproteinases function in activated states. In fact, when there is imbalance in the metalloproteinases and TIMPs activity ratio in favour of metalloproteinases activation, there is increased vascular remodeling and also increased atherosclerotic plaque formation for the arterial side [58].

So, understanding the regulation and the role of metalloproteinases in vascular wall imbalance and in endothelial dysfunction genesis and progression is fundamental for the development of therapeutic agents that may prevent or treat vascular diseases [73].

## 6. Expert Commentary

In order to assess the potential usefulness of the biomarkers in the field of CD and PVD in a way that can be useful for clinicians we can affirm that currently genomic biomarkers can only give information of low to medium risk of developing clinical conditions related to CD and PVD as in this area the participation in the disease is multigenic and it is often difficult to enucleate the contribution of each individual gene, with the exception of the risk of VTE where the genetic alterations identified offer a high level of risk profile.

Transcriptome biomarkers studies are still recent and they have not been reproduced by multiple researchers and the level of risk cannot be assessed at this time yet. Transcriptome biomarkers have the potentiality to measure the kinetic response to a specific effector/drug, to characterize the various stages of development of a disease, but further experiences need to prove their reproducibility.

Proteome biomarkers are currently at a good point of development and are coming to be used in the clinical practice in the near future for both disease predisposition and follow-up tracking during treatment. Some of these such as MMP-9 and NGAL plasma levels have been validated by more than one research group for their effective role in describing response to treatment and their predictive role for some acute complications such as arterial aneurysm rupture.

Table 1 resumes the main biomarkers with a medium/high level of evidence, among those discussed in this paper, whose detection may be turned into clinical practice in the near future.

## 7. Future Perspective and Conclusions

In the next future, it is desirable to develop and validate stratification risk software which may integrate data coming from genetic, transcriptomic, and proteomic biomarkers in order to provide integrated information which could determine effective disease susceptibility, early diagnosis, and staging and tracking of CD and also to provide treatment information at a personalized level.

Thus, a significant proportion of morbidity and mortality related to CD and PVD could be prevented through biomarkers-based strategies, thus making cost-effective interventions accessible and affordable, both for people with established disease and for those at high risk of developing the disease.

## Figures and Tables

**Table 1:** Main biomarkers associated with CD and PVD with a medium/high level of evidence validated by multiple research and detectable through blood samples.

Main disease and subclass	Genomic biomarkers (genes)	Transcriptomic biomarkers	Proteomic biomarkers
CD			
HYT	—	—	MMP-2; MMP-9; MMP-10 (disease development and prognosis)
CAD	—	lncRNA LIPCAR (disease development and prognosis)	—

Arterial PVD			
AAA	LRP1; LDR; SORT1; IL6R MMP-3; AGTR1 (susceptibility)	—	MMP-9; NGAL (disease development and prognosis)
CAA	—	—	—
PAD	—	—	—

Venous PVD			
VV	FOXC2 (susceptibility)	—	—
CVU	—	—	MMP-9; NGAL (disease development, prognosis, response to treatment)
VTE	G20210A Factor V Leiden (susceptibility)	—	MMP-9; NGAL (disease development and prognosis)

## Abbreviations

AAA: Abdominal aortic aneurysm
ADAM: A disintegrin and metalloproteinase
ADAMTS: A disintegrin and metalloproteinase with thrombospondin motifs
AF: Atrial fibrillation
AGTR1: Angiotensin II Receptor, Type 1
ARVC: Arrhythmogenic right ventricular cardiomyopathy
BP: Bblood pressure
CAA: Carotid artery atherosclerosis
CAD: Coronary artery disease
CCL2: Monocyte chemoattractant protein-1
CD: Cardiovascular disease
cDNA: Complementary DNA
COL1A2: Alpha-2 type I collagen gene
CR: Calorie restriction
CVD: Chronic Venous Disease
CVI: Chronic Venous Insufficiency
CVU: Chronic Venous Ulceration
DVT: Deep vein thrombosis
ECM: Extracellular matrix
GWAS: Genome-wide association studies
HDL: High density lipoprotein
HF: Heart failure
HYT: Hypertension
IA: Intracranial aneurysm
ICAM1: Intercellular adhesion molecule 1
ICAS: Internal carotid artery stenosis
IL: Interleukin
IL6R: Interleukin-6 receptor
LDLR: Low density lipoprotein receptor
lncRNA: Long noncoding RNA
LPA: Lipoprotein(a) (LPA)
LRP1: Lipoprotein receptor-related protein 1
LV: Left ventricle
MIF: Migration inhibitory factor
MIP-1*α*/CCL3: Macrophage inflammatory protein-1*α*/CCL3
miRNA: MicroRNA
MMP: Matrix Metalloproteinase
NGAL: Neutrophil gelatinase-associated lipocalin
NMP1: Nucleophosmin-1
PAD: Peripheral arterial disease
PE: Pulmonary embolism
PVD: Peripheral vascular disease
RV: Right ventricle
RVT: Recurrent venous thromboembolism
SELE: E-selectin
SMAD2: Small mother against decapentaplegic 2
SNPs: Coronary artery disease
SORT1: Sortilin 1
SVT: Single venous thromboembolism
TGFB: Transforming Growth Factor
TGFB2: Transforming Growth Factor Beta 2
TGFBR1: Transforming Growth Factor Beta Receptor 1
TGFBR2: Transforming Growth Factor Beta Receptor 2
TIMPS: Tissue Inhibitors of Metalloproteinases
VTE: Venous thromboembolism
VV: Varicose Veins.
